# Beyond Control: Temperature Burden in Patients with Spontaneous Subarachnoid Hemorrhage—An Observational Study

**DOI:** 10.1007/s12028-024-02022-1

**Published:** 2024-06-20

**Authors:** Verena Rass, Bogdan-Andrei Ianosi, Anna Lindner, Philipp Kindl, Alois J. Schiefecker, Raimund Helbok, Bettina Pfausler, Ronny Beer

**Affiliations:** 1 Neurological Intensive Care Unit, Department of Neurology, Medical University of Innsbruck, Anichstraße 35, 6020 Innsbruck, Austria; 2 Department of Neurology, Johannes Kepler University Linz, Linz, Austria

**Keywords:** Subarachnoid hemorrhage, Fever, Temperature management, Spontaneous hypothermia, Functional outcome

## Abstract

**Background:**

Temperature abnormalities are common after spontaneous subarachnoid hemorrhage (SAH). Here, we aimed to describe the evolution of temperature burden despite temperature control and to assess its impact on outcome parameters.

**Methods:**

This retrospective observational study of prospectively collected data included 375 consecutive patients with SAH admitted to the neurological intensive care unit between 2010 and 2022. Daily fever (defined as the area over the curve above 37.9 °C multiplied by hours with fever) and spontaneous hypothermia burden (< 36.0 °C) were calculated over the study period of 16 days. Generalized estimating equations were used to calculate risk factors for increased temperature burdens and the impact of temperature burden on outcome parameters after correction for predefined variables.

**Results:**

Patients had a median age of 58 years (interquartile range 49–68) and presented with a median Hunt & Hess score of 3 (interquartile range 2–5) on admission. Fever (temperature > 37.9 °C) was diagnosed in 283 of 375 (76%) patients during 14% of the monitored time. The average daily fever burden peaked between days 5 and 10 after admission. Higher Hunt & Hess score (*p* = 0.014), older age (*p* = 0.033), and pneumonia (*p* = 0.022) were independent factors associated with delayed fever burden between days 5 and 10. Increased fever burden was independently associated with poor 3-month functional outcome (modified Rankin Scale 3–6, *p* = 0.027), poor 12-month functional outcome (*p* = 0.020), and in-hospital mortality (*p* = 0.045), but not with the development of delayed cerebral ischemia (*p* = 0.660) or intensive care unit length of stay (*p* = 0.573). Spontaneous hypothermia was evident in the first three days in patients with a higher Hunt & Hess score (*p* < 0.001) and intraventricular hemorrhage (*p* = 0.047). Spontaneous hypothermia burden was not associated with poor 3-month outcome (*p* = 0.271).

**Conclusions:**

Early hypothermia was followed by fever after SAH. Increased fever time burden was associated with poor functional outcome after SAH and could be considered for neuroprognostication.

**Supplementary Information:**

The online version contains supplementary material available at 10.1007/s12028-024-02022-1.

## Introduction

Neurogenic and infectious fevers are common in patients with spontaneous subarachnoid hemorrhage (SAH) during the intensive care unit (ICU) stay [1]. Previous studies suggest a link between fever and higher rates of delayed cerebral ischemia (DCI) and poor functional outcomes after SAH [1–5], with a dose-dependent effect of the duration and extent of temperature elevation [6, 7]. Cerebral changes associated with fever include increased metabolic demand [8, 9], increased local oxygen consumption, release of excitatory neurotransmitters and oxygen free radicals [10], ischemic cortical depolarizations [11, 12], increased intracranial pressure, and blood–brain barrier disruption [13], all of which contribute to secondary brain injury. Therefore, fever prevention or controlled normothermia has been suggested as a promising neuroprotective concept to prevent secondary brain injury and improve functional outcome after SAH [14]. However, definitions of fever vary among studies, and the optimal target temperature is not yet known [15]. In a case–control study, 40 patients with SAH underwent advanced fever control (target temperature 37 °C) with surface cooling devices and were compared with 80 controls. Patients with controlled normothermia had a lower temperature burden, a trend toward a lower rate of poor outcome at 3 months, and a statistically significant lower rate of poor outcome at 12 months in multivariable analysis [16]. The recent INTREPID (Impact of Fever Prevention in Brain-Injured Patients) trial [17], a prospective randomized multicenter trial, evaluated the effect of prophylactic normothermia (target temperature 37 °C) achieved with surface cooling compared with standard care fever prevention (temperature < 38.0 °C) on functional outcome after acute brain injury. Final results are pending.

In the current study, we aimed to (1) describe the temporal evolution of the temperature burden in patients with SAH who were treated for fever (target temperature < 38.0 °C), (2) evaluate the impact of temperature burden on outcome, and (3) assess risk factors for spontaneous hypothermia burden, as well as for early and delayed fever burden. We hypothesized that increased fever burden would be associated with poor functional outcome.

## Materials and Methods

### Study Design, Setting, and Patient Selection

The study design was guided by the Strengthening the Reporting of Observational Studies in Epidemiology statement for observational studies. This was a retrospective analysis of prospectively collected data of patients with spontaneous SAH who were admitted to the neurological ICU of a tertiary care hospital (Medical University of Innsbruck) between 2010 and 2022. Inclusion criteria were (1) diagnosis of a spontaneous SAH confirmed by computed tomography (CT) scan or lumbar puncture, regardless of whether an aneurysm was found; (2) 18 years of age or greater; (3) ICU stay of more than 24 h; and (4) temperature monitoring.

The conduct of the study was approved by the local ethics committee (Medical University of Innsbruck, AM4091-292/4.6). The study was conducted in accordance with the Declaration of Helsinki of 1975. Written informed consent was obtained in accordance with local regulations.

### Patient Management and Grading

Patients were treated according to international guidelines [18, 19], with the exception of nimodipine, which was administered intravenously in poor-grade patients. On admission, patients were clinically graded using the Hunt & Hess (H&H) score [20]. Admission CT scans were graded using the modified Fisher score [21] and Hijdra intraventricular sum score [22]. Ruptured aneurysms were secured by clipping or coiling after an interdisciplinary discussion. Transcranial color-coded duplex sonography (LOGIQ S8; GE Healthcare, Chicago, IL) was performed regularly to screen patients for large-vessel vasospasm. Vasospasm was defined as an elevation of mean velocities greater than 120 cm/s in the middle or anterior cerebral artery or a daily change in mean velocities greater than 50 cm/s. In the setting of severe vasospasm, intraarterial nimodipine was administered after confirmation by catheter cerebral angiogram. DCI was diagnosed in the setting of clinical deterioration with a new focal neurologic deficit, a decrease of greater than or equal to two points on the Glasgow Coma Scale, or a new infarct on the CT or magnetic resonance imaging scan not attributable to other causes [23]. In unconscious patients, deterioration of multimodal neuromonitoring parameters (cerebral tissue oxygen tension, cerebral metabolism) was also considered for the diagnosis of DCI [24].

### Temperature Management

Fever control (target temperature < 38.0 °C) was aimed for in all patients. Based on clinical need (i.e., increased intracranial pressure, severe vasospasm), controlled normothermia was used with a target temperature of 36.1–37.5 °C. Mild hypothermia (target temperature 35–36 °C) was used as a third-line escalation therapy for refractory intracranial hypertension in individual patients. An institutional fever protocol following a stepwise approach was used: (1) pharmacological treatment with paracetamol (500–1000 mg iv or po, maximum [max.] 3000 mg/day), and/or diclofenac (50–75 mg iv or po, max. 150 mg/day), and/or naproxen (500 mg po; max. 1000 mg/d), and/or metamizole (1000 mg iv or po; max. 4000 mg/d), and/or pethidine (100 mg iv, max. 300 mg/d) together with physical methods including surface cooling with peppermint tea and cold infusions; (2) in pharmacologically refractory fever, the use of a feedback devices (surface cooling device, Arctic Sun; endovascular cooling device, Thermogard XP) was chosen according to the treating neurointensivist’s discretion. A feedback device was the first choice in individual patients in whom controlled normothermia or mild hypothermia was sought (i.e., in the setting of early severe intracranial hypertension). Antipyretics were infused slowly over 30–60 min to minimize associated blood pressure drops [25].

In the presence of fever, a diagnostic workup for a potential infectious cause was performed in all patients. Infectious complications were diagnosed according to predefined criteria set forth by the Centers for Disease Control and Prevention criteria [26]. Temperature was continuously recorded by temperature-sensing indwelling urinary catheters.

### Data Collection

Patient demographics, hospital complications and outcomes were prospectively collected and discussed in weekly meetings between the study team and attending neurointensivists. Temperature measurements were stored in an electronic patient data management system (CentricityTM Critical Care 8.1 SP7; GE Healthcare Information Technology, Dornstadt, Germany) at a granularity of 3 min to 1 h. For the purpose of this study, mean values over one hour were calculated after data cleaning for artifacts (< 34 °C and > 42 °C or implausible values). Time and dose of antipyretics were also recorded in the electronic patient data management system. Functional neurological outcome was assessed by telephone interview at 3 months and by face-to-face visits at 12 months using the modified Rankin Scale score (mRS).

### Study Outcome Measures

The primary outcome measure was the daily fever burden, defined as the area over the curve above 37.9 °C multiplied by hours with fever and the spontaneous hypothermia burden (< 36.0 °C), during the 16 days after admission. The key secondary outcome end point was functional outcome at 3 months after SAH using the mRS, with poor outcome defined as mRS score 3–6.

### Data Management and Statistical Analysis

Based on the available data points of temperature measurements (Supplemental Fig. 1), the first 16 days after ICU admission were defined as the study period. The first 24 h after ICU admission was defined as day 1.

Variables are presented as counts with percentages, medians with interquartile ranges (IQRs), or means ± standard deviations, as appropriate. Continuous variables were tested for normality and compared using the *t*-test or Mann–Whitney *U*-test. Differences between binary variables were analyzed using the Fisher’s exact test. Hourly temperature values were linearly interpolated if a maximum of three consecutive hourly values were missing (6,824/79,913 interpolated values, 8.5%). Patient days were excluded if > 30% of temperature values per day were missing after interpolation (requirement of ≥ 17 available temperature values per 24 h). The daily fever burden (°C × hours) was calculated by subtracting each patient’s hourly recorded temperature from 37.9 °C and summarized for each day. If the actual temperature was below 37.9 °C, a value of 0 was assigned. Mean (± standard deviation) fever burden was normalized to measurements per day (daily fever burden/daily measured hours × 24 h). Identically, temperature burdens were calculated for temperatures < 36.0 °C, > 37.0 °C, > 37.5 °C, and > 38.2 °C. For the calculation of the spontaneous hypothermia burden < 36.0 °C, days with the use of feedback devices (i.e., surface and endovascular) were excluded. If patients had less than 16 days of temperature records, they were included in the analysis through that day.

To identify risk factors for fever/hypothermia burden, multivariable logistic regression analysis with generalized estimating equation models was used to account for repeated measures within a patient over the study period [27]. Early burden (days 1–3) represents the time of early brain injury, and the delayed burden (days 5–10) is based on the peak optical fever burden. Generalized estimating equation models were also used to evaluate the impact of temperature burden on variable outcome parameters. Multivariable models were adjusted for important predefined covariates (H&H score on admission; age; cumulative daily doses of paracetamol, metamizole, naproxen, diclofenac, and pethidine, whether used as antipyretics or for pain; and use of surface/endovascular cooling per day). Outcome models were also adjusted for infections (pneumonia, sepsis/bacteremia, ventriculitis, urinary tract infection) and DCI. Clinically meaningful and significantly associated factors (*p* < 0.1) in the univariate analysis were entered into a multivariable logistic regression model along with predefined covariates. Backward elimination was used to remove nonsignificant factors until the best model with the lowest Quasilikelihood under the Independence model Criterion values were obtained and the predictors were significant (*p* < 0.05). A *p* value less than 0.05 was considered statistically significant. Statistical analysis was performed with IBM SPSS Statistics version 24 64-bit edition.

## Results

### Study Population and Clinical Characteristics

Of 483 patients, 375 patients with > 70% of hourly temperature measurements available for at least one day within the study period were included (Supplemental Fig. 2). A total of 3,439 days were analyzed, representing a median of 10 days (IQR 3–15) per patient. Table 1 shows the difference between included and excluded patients. Included patients had a higher disease severity and worse outcome measures. However, all severity grades were represented (Table 1) with a median H&H score of 3 (2–5). Patients were 58 (IQR 49–68) years of age. Vasospasm was diagnosed in 52% of patients, and 21% developed DCI. Seventeen percent of patients died in the hospital, and the median 3-month mRS was 2 (IQR 1–5). Supplemental Table 1 shows demographics and hospital complications for patients with good and poor 3-month outcomes.

**Table 1:** Study participant demographics, baseline characteristics, hospital complications, and outcomes

	Included patients (*N* = 375)	Excluded patients (*N* = 108)	*P*-value^a^
Baseline characteristics			
Age	58 (49–68)	54 (48–63)	0.144
Female sex	240 (64)	53 (49)	**0.007**
Arterial hypertension	160 (43)	26 (24)	** < 0.001**
Smoking history	138 (37)	24 (23)	**0.007**
Admission variables			
Admission Hunt & Hess score			
1	82 (22)	67 (62)	** < 0.001**
2	68 (18)	20 (19)	
3	79 (21)	5 (5)	
4	34 (9)	0 (0)	
5	112 (30)	16 (15)	
Loss of consciousness at onset	166 (44)	18 (17)	** < 0.001**
Modified Fisher Score on admission	4 (3–4)	2 (1–3)	** < 0.001**
Intraparenchymal bleeding	81 (22)	11 (10)	**0.008**
SEBES score	1 (0–3)	0 (0–1)	** < 0.001**
Hijdra intraventricular sum score	3 (0–6)	0 (0–3)	** < 0.001**
Aneurysm detected	318 (85)	43 (40)	** < 0.001**
Aneurysm treatment			
Coiling	205 (55)	27 (25)	** < 0.001**
Clipping	102 (27)	3 (3)	
No intervention (no aneurysm)	55 (15)	69 (64)	
No intervention (withhold therapy)	13 (4)	9 (8)	
Withdrawn therapy during ICU stay	31 (8)	5 (5)	0.297
Hospital complications			
Hydrocephalus requiring external ventricular drain	213 (57)	9 (8)	** < 0.001**
Mechanical ventilation during ICU stay	328 (88)	36 (33)	** < 0.001**
Ventilated days	8 (1–19)	0 (0–1)	** < 0.001**
Large-vessel vasospasm	195 (52)	16 (15)	** < 0.001**
Delayed cerebral ischemia	78 (21)	5 (5)	** < 0.001**
Sepsis/bacteremia	57 (15)	1 (1)	** < 0.001**
Pneumonia	168 (45)	5 (5)	** < 0.001**
Ventriculitis	49 (13)	0 (0)	** < 0.001**
Urinary tract infection	98 (26)	14 (13)	**0.004**
Outcomes			
ICU length of stay, days	19 (11–31)	8 (3–12)	** < 0.001**
Hospital mortality	65 (17)	21 (20)	0.669
3-month functional outcome (modified Rankin Scale)^b^			
0	62 (17)	45 (42)	** < 0.001**
1	76 (21)	30 (28)	
2	52 (14)	10 (9)	
3	32 (9)	0 (0)	
4	28 (8)	0 (0)	
5	37 (10)	0 (0)	
6	74 (21)	22 (21)	

Data are given in *n* (%) or median (IQR)

Bold numbers signify statistical differences

*ICU* intensive care unit, *IQR* interquartile range, *SEBES* Subarachnoid Hemorrhage Early Brain Edema Score

^a^Differences across the included and excluded patients were calculated with the Mann–Whitney *U*-test, *t*-test, or the Fisher’s exact test, as appropriate

^b^15 patients were lost to 3-month follow-up

### Timely Evolution of Temperature Burden

**Fig. 1:**
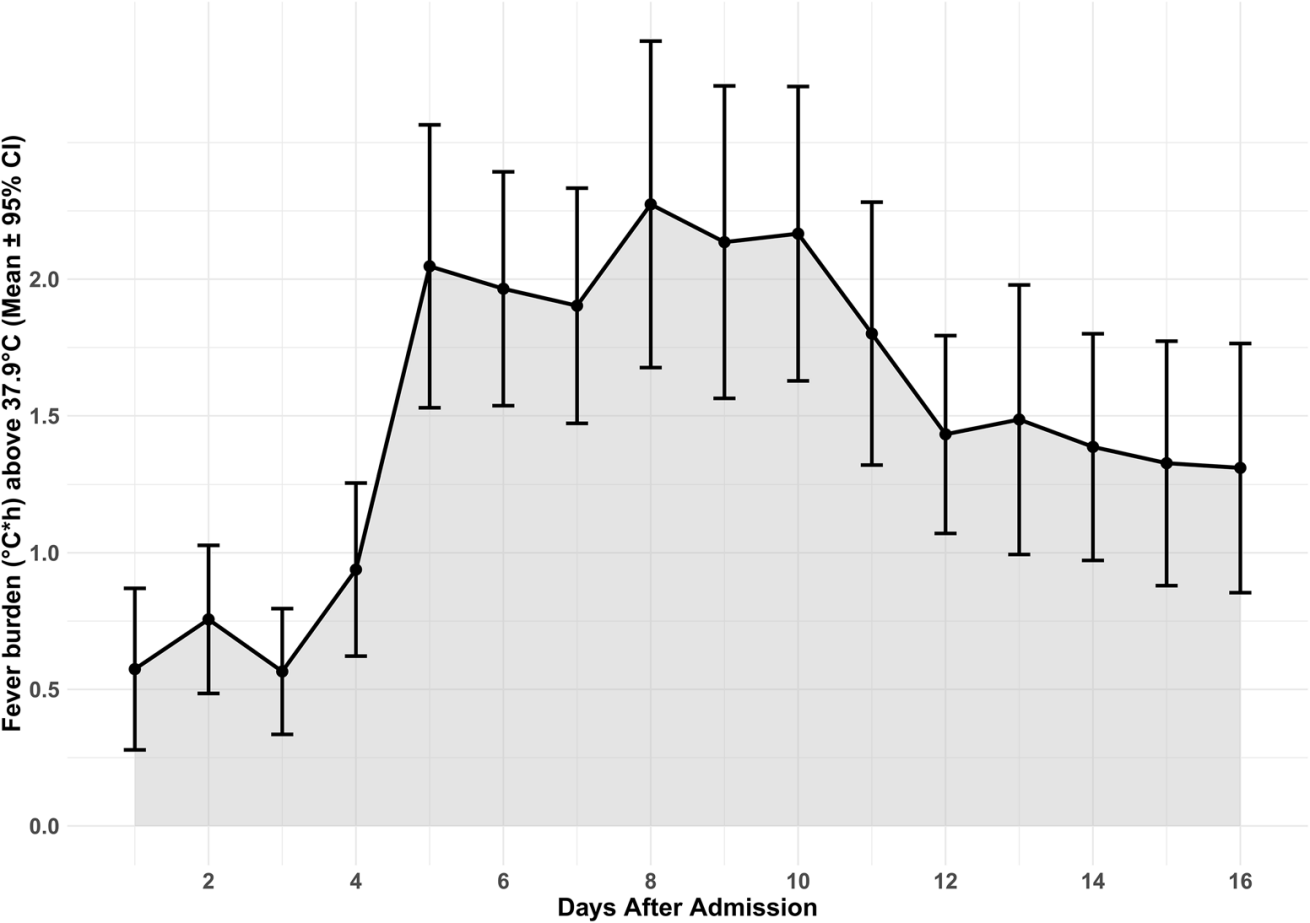
Average daily fever burden. The daily fever burden, defined as the mean (95% CI) area over the curve of fever > 37.9 °C (equals the sum of depth of abnormalities multiplied by the hours spent in fever > 37.9 °C normalized to monitored time) is reported in °C × hours. CI, confidence interval

The mean temperature per patient during the study period was 37.05 ± 0.55 °C (Supplemental Fig. 1). Fever (> 37.9 °C) developed in 283/375 (76%) patients on 14% of the monitored study days. The average daily fever burden (> 37.9 °C) is shown in Fig. 1, with a peak between days 5 and 10. Supplemental Fig. 3 depicts the temperature burden for > 37.0 °C, > 37.5 °C, and > 38.2 °C. The average daily hours spent with fever (> 37.9 °C) was 3.4 ± 5.6 h, with a peak at days 5–10 (4.5 ± 6.1 h; Supplemental Fig. 4). Surface and endovascular cooling devices were used in 33 and 28 patients, respectively on 439/3439 (13%) study days. Therapeutic mild hypothermia was used in six patients during 32 cumulative study days. Supplemental Fig. 5 shows the average daily fever burden in patients with and without feedback devices on their respective days.

**Fig. 2:**
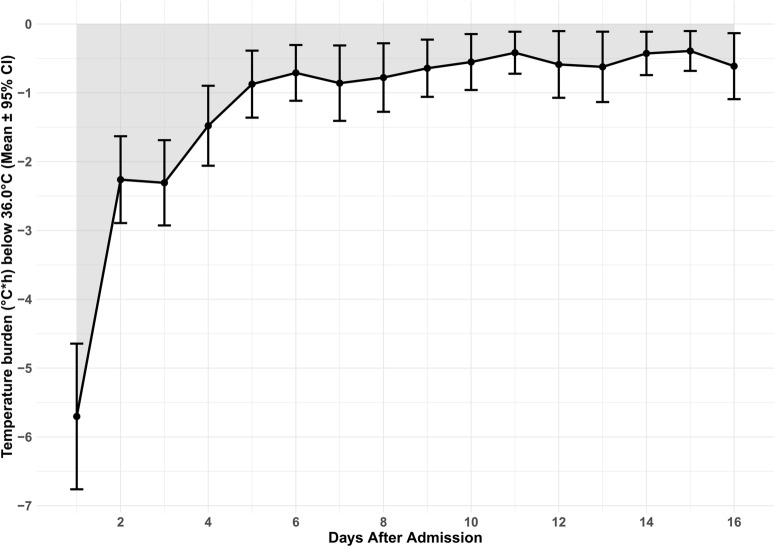
Average daily spontaneous hypothermia burden. The daily spontaneous hypothermia burden, defined as the mean (95% CI) area under the curve < 36.0 °C (equals the sum of depth of abnormalities multiplied by the hours spent in hypothermia < 36.0 °C normalized to monitored time) is reported in °C × hours. Days with use of feedback devices were excluded. CI, confidence interval

Spontaneous hypothermia < 36.0 °C occurred in 70% (259/371) of the patients during 9% of the monitored time, especially during the first 3 days after admission, excluding the days with feedback devices. The area under the curve < 36 °C represents a temperature burden nadir within the first 3 days after SAH (Fig. 2).

### Factors Associated with Early Temperature Burden

Factors independently associated with a higher spontaneous hypothermia burden < 36 °C within the first 3 days, included higher H&H score (adjusted odds ratio [adjOR] = 0.46, 95% confidence interval [CI] 0.33–0.65, *p* < 0.001) and intraventricular hemorrhage on admission (adjOR = 0.44, 95% CI 0.20–0.99, *p* = 0.047; Supplemental Table 2).

Admission variables independently associated with higher early fever burden (> 37.9 °C) within the first 3 days included hydrocephalus requiring an external ventricular drainage (adjOR = 2.08, 95% CI 1.20–3.61, *p* = 0.009) and Hijdra intraventricular sum score ≥ 3 (adjOR = 1.65, 95% CI 1.05–2.60, *p* = 0.029; Supplemental Table 3).

### Factors Associated with Delayed Fever Burden

Among admission factors and hospital complications, higher H&H score (adjOR = 1.32, 95% CI 1.06–1.64, *p* = 0.014), older age (adjOR = 1.03, 95% CI 1.002–1.058, *p* = 0.033) and presence of pneumonia (adjOR = 2.18, 95% CI 1.12–4.25, *p* = 0.022) were independent factors associated with delayed fever burden between days 5 and 10 (Supplemental Table 4).

### Impact of Temperature Burden on Outcome Measures

**Fig. 3:**
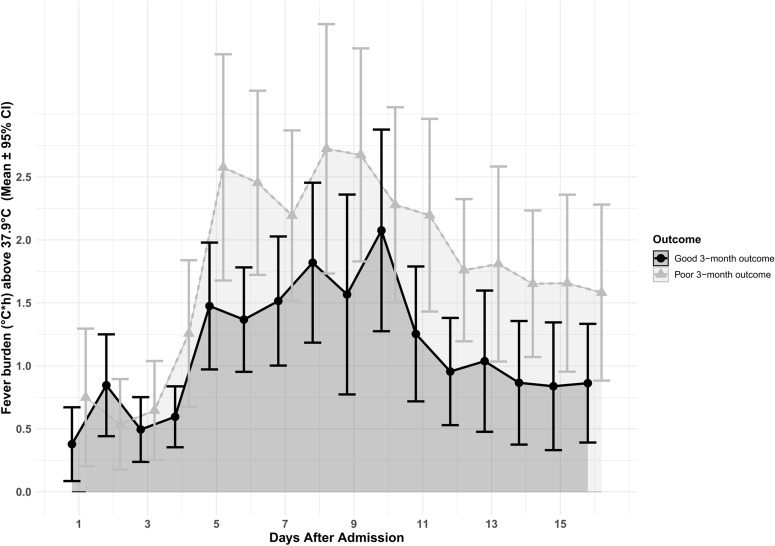
Average daily fever burden (> 37.9 °C; in °C × hours) for patients with good (modified Rankin Scale score 0–2) and poor (modified Rankin Scale score 3–6) functional outcome 3 months after subarachnoid hemorrhage. CI, confidence interval

At 3 and 12 months, 14 and 28 (out of 375) patients, respectively, were lost to follow-up. Greater fever burden (> 37.9 °C) was independently associated with poor 3-month functional outcome (*p* = 0.027; Tables 2 and 3, Fig. 3), worse 3-month outcome (*p* < 0.001; mRS as ordinal dependent variable), poor 12-month functional outcome (*p* = 0.020), and in-hospital mortality (*p* = 0.045) in models corrected for known outcome predictors, infections, and daily sum of pharmacologic or daily physical fever treatments used. Similar results were obtained for fever burden with a cutoff of > 38.2 °C, but not > 37.0 °C. A fever burden > 37.5 °C was only associated with a worse 3-month outcome (*p* = 0.012), but not with the dichotomized outcome (*p* = 0.155) (Table 3). Fever burden was not associated with the development of DCI (*p* = 0.660; Table 3) or ICU length of stay (*p* = 0.573). Maximum temperature per day or occurrence of fever > 37.9 per day was not associated with poor 3-month functional outcome (*p* > 0.05, Table 3).

**Table 2:** Independent impact of daily fever burden (> 37.9 °C) within the study period of 16 days on poor 3-month functional outcome in 361 patients

Variables	Adjusted OR	95%-CI	*P-*value
Fever burden > 37.9 °C, °C × h	1.07	1.01–1.32	0.027
Admission Hunt & Hess score	1.64	1.28–2.09	< 0.001
Age, years	1.06	1.04–1.09	< 0.001
Delayed cerebral ischemia	2.79	1.33–5.85	0.007
Sepsis/bacteremia	3.07	1.33–7.10	0.009
Pneumonia	1.42	0.78–2.57	0.253
Urinary tract infection	1.33	0.67–2.64	0.418
Ventriculitis	0.87	0.41–1.87	0.722
Diclofenac, daily sum (mg)	0.995	0.991–0.999	0.010
Naproxen, daily sum (mg)	0.999	0.998–1.001	0.332
Paracetamol, daily sum (mg)	1.00	1.00–1.00	0.163
Metamizole, daily sum (mg)	1.00	0.99–1.00	0.211
Pethidin, daily sum (mg)	0.999	0.995–1.003	0.621
Treatment with feedback device, per day	1.60	0.80–3.19	0.180

Multivariable logistic regression analysis was done with a generalized estimating equation model with an independent correlation matrix to account for repeated measures; the dependent variable (poor 3-month outcome, modified Rankin Scale score 3–6) was used as binary variable. The model was corrected for known outcome predictors (Hunt & Hess score, age, and delayed cerebral ischemia), infections as well as daily sums of used pharmacologic or physical fever treatment. Respective OR (95%-CI) are given

CI, confidence interval, OR, odds ratio

**Table 3:** Impact of daily fever burden within the study period of 16 days on outcome parameters in 347 to 375 patients

	Adjusted OR	95% -CI	*P-*value
Models: development of DCI^a^			
Fever burden > 37.9 °C, °C × h	1.01	0.97–1.06	0.660
Fever burden > 37.0 °C, °C × h	1.01	0.99–1.03	0.308
Fever burden > 37.5 °C, °C × h	1.01	0.98–1.04	0.448
Fever burden > 38.2 °C, °C × h	1.02	0.95–1.08	0.651
Models: poor functional 3-month outcome (mRS 3–6)^b^			
Fever burden > 37.9 °C, °C × h	1.07	1.01–1.32	0.027
Fever burden > 37.0 °C, °C × h	1.00	0.98–1.02	0.883
Fever burden > 37.5 °C, °C × h	1.03	0.99–1.06	0.155
Fever burden > 38.2 °C, °C × h	1.12	1.01–1.24	0.037
Maximum temperature per day, °C	0.919	0.724–1.17	0.489
Maximum temperature per day > 37.9 °C	1.04	0.73–1.47	0.851
Models: functional worse 3-month outcome, ordinal^b^			
Fever burden > 37.9 °C, °C × h	1.08	1.03–1.13	< 0.001
Fever burden > 37.0 °C, °C × h	1.01	0.99–1.03	0.299
Fever burden > 37.5 °C, °C × h	1.03	1.01–1.06	0.012
Fever burden > 38.2 °C, °C × h	1.13	1.05–1.21	0.001
Maximum temperature per day, °C	0.96	0.80–1.16	0.691
Maximum temperature per day > 37.9 °C	1.11	0.86–1.43	0.422
Models: poor functional 12-month outcome (mRS 3–6)^c^			
Fever burden > 37.9 °C, °C × h	1.07	1.01–1.13	0.020
Fever burden > 37.0 °C, °C × h	0.99	0.97–1.02	0.494
Fever burden > 37.5 °C, °C × h	1.02	0.99–1.06	0.229
Fever burden > 38.2 °C, °C × h	1.12	1.02–1.24	0.018
Models: hospital mortality^a^			
Fever burden > 37.9 °C, °C × h	1.07	1.00–1.14	0.045
Fever burden > 37.0 °C, °C × h	0.997	0.97–1.03	0.865
Fever burden > 37.5 °C, °C × h	1.03	0.98–1.07	0.246
Fever burden > 38.2 °C, °C × h	1.11	1.01–1.21	0.037
Models: ICU length of stay, days^a^			
Fever burden > 37.9 °C, °C × h	1.09	0.81–1.47	0.573
Fever burden > 37.0 °C, °C × h	1.03	0.91–1.16	0.665
Fever burden > 37.5 °C, °C × h	1.04	0.87–1.25	0.647
Fever burden > 38.2 °C, °C × h	1.12	0.72–1.76	0.615

Multivariable logistic regression analysis was done with a generalized estimating equation model with an independent correlation matrix to account for repeated measures; the dependent variable was used as binary (development of DCI, dichotomized 3-month and 12-month outcome, hospital mortality), ordinal (3-month mRS) or linear (ICU days) variable. For each fever cutoff (i.e., > 37.9 °C, > 37.0°C, > 37.5 °C, > 38.2 °C) separate models were calculated. All models were corrected for Hunt & Hess score on admission, age, daily doses of paracetamol, metamizole, naproxen, diclofenac, pethidine, daily usage of surface/intravascular cooling, pneumonia, ventriculitis, sepsis/bacteremia, urinary tract infection, and DCI (except for the DCI model)

*CI* confidence interval, *DCI* delayed cerebral ischemia, *ICU* intensive care unit, *mRS* modified Rankin Scale, OR, odds ratio

^a^375 patients

^b^14 patients were lost to 3-month follow-up, resulting in 361 included patients

^c^28 patients were lost to 12-month follow-up, resulting in 347 included patients

Spontaneous hypothermia burden was not associated with DCI (*p* = 0.526) or poor 3-month outcome (*p* = 0.271).

## Discussion

The main findings of the current study are that (1) spontaneous hypothermia is common in the early phase after SAH and is followed by an increased fever burden in the delayed phase, and (2) an increased fever burden is associated with poor outcome in a group of patients with strict temperature control. Intraventricular hemorrhage was linked to both early fever and early hypothermia burden. Increased disease severity, age, and pneumonia were associated with a delayed peak of fever burden.

Our data reflect a real-world clinical situation with a large population of patients with SAH of all severity grades in whom temperatures were measured regularly, providing high granularity data to express temperature burdens. Importantly, all results should be interpreted considering that fever prevention was a therapeutic goal using a stepwise protocol. Similar to our local procedures, recent consensus recommendations for targeted temperature management (TTM) in patients with hemorrhagic and ischemic stroke requiring ICU admission suggest that ideally, core temperature should be continuously measured and maintained between 36.0 and 37.5 °C [14]. TTM should be initiated promptly upon detection of fever using a local TTM protocol to improve patient outcomes [14].

Our findings add to previous work by demonstrating two stages of temperature abnormalities after SAH. Specifically, in poor-grade patients with intraventricular blood, temperature spontaneously dropped to < 36.0 °C in the early phase after SAH, consistent with previous studies [28, 29]. Spontaneous hypothermia may be a consequence of increased brain damage involving hypothalamic and brainstem areas responsible for temperature control. Interestingly, we found that an increased amount of intraventricular hemorrhage was also associated with an early fever burden, suggesting that intraventricular hemorrhage leads to temperature dysregulation with resulting increased temperature variability. A possible explanation is a central change in the temperature set point due to toxic and inflammatory effects of the blood on the brainstem and hypothalamus [2, 30].

We found a shift to hyperthermic temperature levels, peaking at 5 to 10 days after SAH, which was associated with primary injury severity, age, and pneumonia. Fever may therefore be a marker of injury severity [2, 6] as well as of infection in poor-grade patients with a more complicated course requiring mechanical ventilation. Consistent with the literature [2], we could only identify pneumonia as a factor associated with delayed infectious fever.

Data remain heterogenous regarding the definition of fever, resulting in variations between studies ranging from 37.0 to 38.3 °C [4, 6, 16, 31, 32]. Current SAH guidelines are vague and do not specify a target temperature threshold [15, 19] whereas recent consensus recommendations advocate maintaining temperatures between 36.0 and 37.5 °C in patients with SAH [14]. Therefore, we provide several cutoffs and found that temperature burdens > 37.9 °C and > 38.2 °C were similar in terms of outcome prediction; however, temperature burdens > 37.0 °C and > 37.5 °C did not discriminate patients with poor short-term and long-term outcomes. We found that the maximum temperature per day or the daily occurrence of fever (> 37.9 °C) was not associated with poor outcome. This suggests that a fever burden, including the depth and time of exposure, is more useful in determining outcome than a single threshold.

In contrast to previous studies, we did not find a link between fever burden and the occurrence of DCI [33]. This is interesting because fever has been shown to be associated with a higher incidence of cortical spreading depolarizations [11, 12]. Clusters of cortical spreading depolarizations can lead to infarction and DCI [34]. It is possible that we did not find an association because vigilant temperature monitoring and consequent fever prophylaxis were used, especially in patients at high risk for DCI.

To date, it is unclear whether treatment of fever or controlled normothermia improves outcomes after SAH [15]. Although fever treatments are effective in controlling temperature, there is no evidence that either modality improves outcomes. Observational studies suggest a strong link between (refractory) fever and worse outcomes after SAH, which is supported by our data. Therefore, we are faced with two questions: (1) Are the beneficial effects of controlled normothermia/fever prevention outweighed by associated complications such as shivering, increased duration and level of sedation, treatment related hypotension, and promotion of infectious complications by eliminating the febrile response, and (2) Does fever cause worse outcomes or is it rather an epiphenomenon of worse severity of primary and secondary brain injury? From a pathophysiological perspective, fever increases brain metabolism and exacerbates neuronal injury by multiple mechanisms, potentially worsening early and secondary brain injury [24, 35]. This is supported by the fact that several studies, including our current data, have shown that the negative effect of fever persists consistently even after controlling for other predictors of poor outcome [2–4, 6]. Importantly, the effect of increased fever burden remained for poor 12-month outcome after completion of rehabilitation in our cohort. This finding is novel compared with other studies that have mainly examined outcome at discharge [4, 5] or at 3 months [1, 2, 6]. Finally, a gap in knowledge remains regarding the optimal target temperature and duration of fever prevention.

In clinical practice and for future guidelines, the current study may help to select those patients in whom vigilant fever monitoring and appropriate treatment are of utmost importance to improve outcomes and mitigate secondary brain injury. These patient-specific factors include intraventricular hemorrhage, poor-grade SAH, advanced age, and pneumonia. Based on an individualized approach, early escalation to feedback devices may be reasonable in this patient population to effectively prevent fever [14].

There are several limitations that merit consideration. First, the observational nature of the study shows associations but not necessarily causality. Second, there is a considerable dropout rate of patients, with a potential selection bias toward worse patients due to the fact that we aimed to present temperature burdens based on a high granularity of data. Nevertheless, all severity groups were included. In addition, we decided to calculate daily temperature burdens to include patients with a shorter ICU stay (good-grade patients or those who died soon), who did not have the opportunity to accumulate large temperature burdens. Third, we performed several regression analyses to evaluate the impact of fever burden (with different temperature cutoffs) on different outcome parameters. Because of the possibility of type I error due to multiple comparisons, the results of analyses other than the primary and key secondary end points should be interpreted as exploratory. Fourth, confounders and associated factors for multivariable models were selected based on clinical relevance (predefined) and significance in univariate analysis. Factors defined by significance tests may depend on the characteristics of a cohort population, reducing the external validity. Fifth, we presented real-world data with a fever prevention approach applied to all patients without a control group.

## Conclusions

Our study shows two phases of temperature abnormalities with spontaneous hypothermia in the early phase followed by a peak of fever burden in the subacute phase after SAH despite rigorous temperature control measures. A higher burden of fever extent and time duration was associated with a poor functional outcome and could be considered for neuroprognostication after SAH.

## Supplementary Information

Below is the link to the electronic supplementary material.

Supplementary file1 (DOCX 1203 KB)
