# Supplementary material for: Beyond Control: Temperature Burden in Patients with Spontaneous Subarachnoid Hemorrhage—An Observational Study
Source: Neurocrit Care. 2024 Jun 20;41(3):974–84. doi: 10.1007/s12028-024-02022-1 (PMC11599626; doi:10.1007/s12028-024-02022-1)
Supplement: Supplementary file 1 — Supplementary file1 (DOCX 1203 KB) [file 12028_2024_2022_MOESM1_ESM.docx]

**Supplemental digital content**

**TABLES**

| **Supplemental Table 1: Subject demographics, baseline characteristics, and hospital complications for patients with good and poor 3-month outcomes.** | | | | |
| --- | --- | --- | --- | --- |
|  | | Good outcome (mRS 0-2)  N=190 | Poor outcome (mRS 3-6)  N=171 | P-value^a^ |
| **Baseline characteristics** | |  |  |  |
| Age | | 54 (45-60) | 64 (53-73) | **<0.001** |
| Sex, female | | 115 (61) | 117 (68) | 0.125 |
| Arterial hypertension | | 70 (37) | 80 (47) | 0.069 |
| **Admission variables** | |  |  |  |
| Admission Hunt & Hess score |  | 2 (1-3) | 4 (3-5) | **<0.001** |
| Loss of consciousness at onset | | 54 (28) | 105 (61) | **<0.001** |
| Modified Fisher Score on admission | | 3 (2-4) | 4 (3-4) | **<0.001** |
| Intraparenchymal bleeding | | 25 (13) | 55 (32) | **<0.001** |
| SEBES score | | 1 (0-2) | 2 (0-3) | **0.006** |
| Hijdra intraventricular sum score | | 2 (0-4) | 5 (2-7) | **<0.001** |
| **Aneurysm treatment** | |  |  |  |
| Coiling | | 105 (55) | 91 (53) | **<0.001** |
| Clipping | | 44 (23) | 55 (32) |  |
| No intervention (no aneurysm) | | 41 (22) | 12 (7) |  |
| No intervention (withhold therapy) | | 0 (0) | 13 (8) |  |
| Withdrawn therapy during ICU stay | | 0 (0) | 31 (18) | **<0.001** |
| **Hospital complications** | |  |  |  |
| Hydrocephalus requiring external ventricular drain | | 77 (41) | 128 (75) | **<0.001** |
| Mechanical ventilation during ICU stay | | 149 (78) | 167 (98) | **<0.001** |
| Ventilated days | | 2 (1-12) | 16 (6-27) | **<0.001** |
| Large-vessel vasospasm | | 95 (50) | 95 (56) | 0.342 |
| Delayed cerebral ischemia | | 26 (14) | 50 (29) | **<0.001** |
| Sepsis/Bacteremia | | 15 (8) | 39 (23) | **<0.001** |
| Pneumonia | | 62 (33) | 98 (57) | **<0.001** |
| Ventriculitis | | 20 (11) | 28 (16) | 0.121 |
| Urinary tract infection | | 43 (23) | 52 (30) | 0.096 |
| Data are given in n (%) or median (IQR).  ^a^ Differences between patients with good and poor outcomes were calculated with the Mann Whitney U, T-Test, or the Fisher's exact test, as appropriate. Bold numbers signify statistical differences. | | | | |

| **Supplemental Table 2**. Multivariable models identifying risk factors for early spontaneous hypothermia burden (<36.0°C) within 3 days after admission in 309 patients. | | | |
| --- | --- | --- | --- |
|  | **Adjusted OR** | **95%-CI** | **P-value** |
| Admission Hunt & Hess score | 0.46 | 0.33-0.65 | **<0.001** |
| Intraventricular hemorrhage on admission | 0.44 | 0.20-0.99 | **0.047** |
| Age, years | 0.98 | 0.95-1.01 | 0.233 |
| Diclofenac, daily sum (mg) | 1.01 | 1.01-1.02 | <0.001 |
| Naproxen, daily sum (mg) | 1.00 | 0.998-1.00 | 0.708 |
| Paracetamol, daily sum (mg) | 1.00 | 1.00-1.00 | <0.001 |
| Metamizole, daily sum (mg) | 1.00 | 1.00-1.00 | 0.012 |
| Pethidin, daily sum (mg) | 1.02 | 1.01-1.03 | 0.003 |
| Multivariable logistic regression analysis was done with a generalized estimating equation (GEE) model with an independent correlation matrix to account for repeated measures; the dependent variable (hypothermia burden) was used as linear variable. | | | |

| **Supplemental Table 3**. Multivariable models identifying risk factors for early fever burden (>37.9°C) within 3 days after admission in 323 patients. | | | |
| --- | --- | --- | --- |
| **Model 1** | **Adjusted OR** | **95%-CI** | **P-value** |
| Hydrocephalus req. external ventricular drainage | 2.08 | 1.20-3.61 | **0.009** |
| Admission Hunt & Hess score | 1.07 | 0.89-1.29 | 0.498 |
| Age, years | 0.996 | 0.99-1.01 | 0.444 |
| Diclofenac, daily sum (mg) | 1.01 | 1.00-1.01 | 0.011 |
| Naproxen, daily sum (mg) | 0.999 | 0.998-1.000 | 0.088 |
| Paracetamol, daily sum (mg) | 1.00 | 1.001-1.001 | <0.001 |
| Metamizole, daily sum (mg) | 1.00 | 1.000-1.001 | 0.261 |
| Pethidin, daily sum (mg) | 1.02 | 0.99-1.05 | 0.144 |
| Treatment with feedback device, per day | 0.72 | 0.42-1.24 | 0.236 |
| **Model 2** | **Adjusted OR** | **95%-CI** | **P-value** |
| Hijdra intraventricular sum score ≥3^a^ | 1.65 | 1.05-2.60 | **0.029** |
| Admission Hunt & Hess score | 1.09 | 0.92-1.29 | 0.336 |
| Age, years | 0.995 | 0.98-1.01 | 0.366 |
| Diclofenac, daily sum (mg) | 1.00 | 1.00-1.01 | 0.049 |
| Naproxen, daily sum (mg) | 0.999 | 0.997-1.000 | 0.067 |
| Paracetamol, daily sum (mg) | 1.00 | 1.000-1.001 | <0.001 |
| Metamizole, daily sum (mg) | 1.00 | 1.00-1.00 | 0.487 |
| Pethidin, daily sum (mg) | 1.02 | 0.996-1.05 | 0.100 |
| Treatment with feedback device, per day | 0.83 | 0.50-1.34 | 0.457 |
| Multivariable logistic regression analysis was done with a generalized estimating equation (GEE) model with an independent correlation matrix to account for repeated measures; the dependent variable (fever burden) was used as linear variable. Two models were used based on the collinearity between hydrocephalus and Hijdra intraventricular sum score.  ^a^ based on the median Hijdra intraventricular sum score of 3 (IQR, 0-6) | | | |

| **Supplemental Table 4**. Multivariable models identifying risk factors for delayed fever burden (>37.9°C) within days 5-10 days after admission in 297 patients. | | | |
| --- | --- | --- | --- |
| **Variables** | **Adjusted OR** | **95%-CI** | **P-value** |
| Admission Hunt & Hess score | 1.32 | 1.06-1.64 | **0.014** |
| Age, years | 1.03 | 1.002-1.058 | **0.033** |
| Pneumonia | 2.18 | 1.12-4.25 | **0.022** |
| Diclofenac, daily sum (mg) | 1.01 | 1.00-1.01 | 0.001 |
| Naproxen, daily sum (mg) | 0.999 | 0.997-1.000 | 0.006 |
| Paracetamol, daily sum (mg) | 1.00 | 1.00-1.00 | 0.007 |
| Metamizole, daily sum (mg) | 1.001 | 1.000-1.001 | 0.022 |
| Pethidin, daily sum (mg) | 1.03 | 1.01-1.04 | 0.003 |
| Treatment with feedback device, per day | 0.23 | 0.14-0.37 | <0.001 |
| Multivariable logistic regression analysis was done with a generalized estimating equation (GEE) model with an independent correlation matrix to account for repeated measures; the dependent variable (fever burden) was used as linear variable. | | | |

**FIGURES**


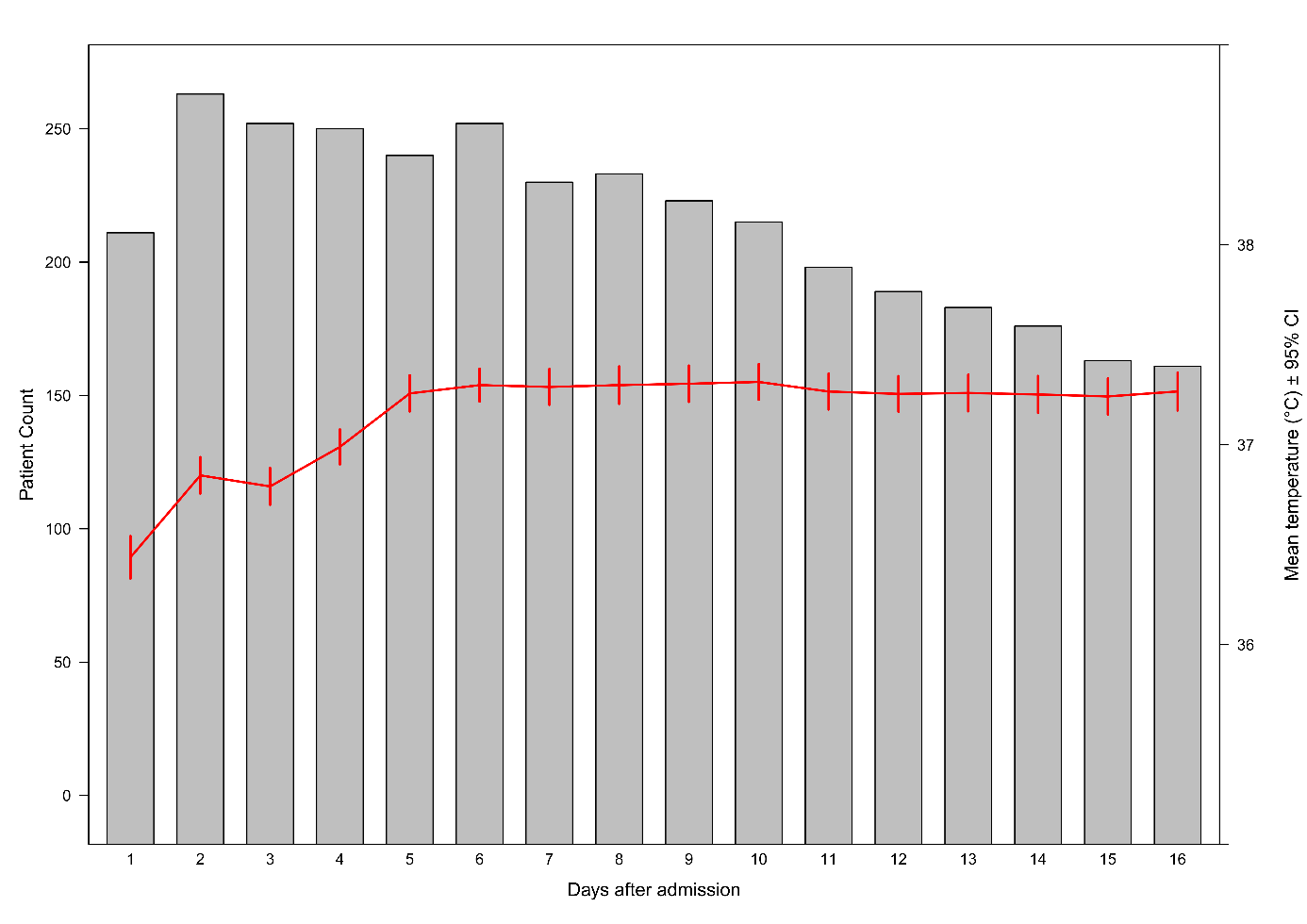


**Supplemental Figure 1:** Daily patient counts and mean (95%-CI) bladder temperatures over the first 16 days after admission in subarachnoid hemorrhage patients.


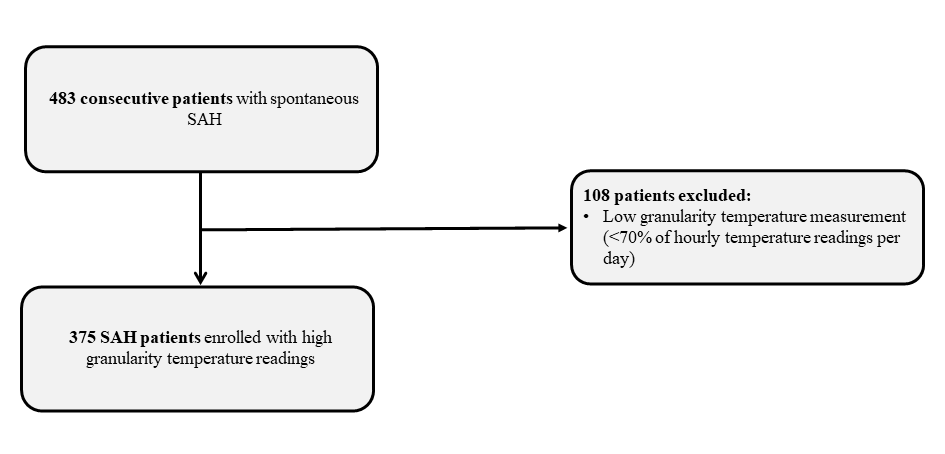


**Supplemental Figure 2:** Patient flow chart.


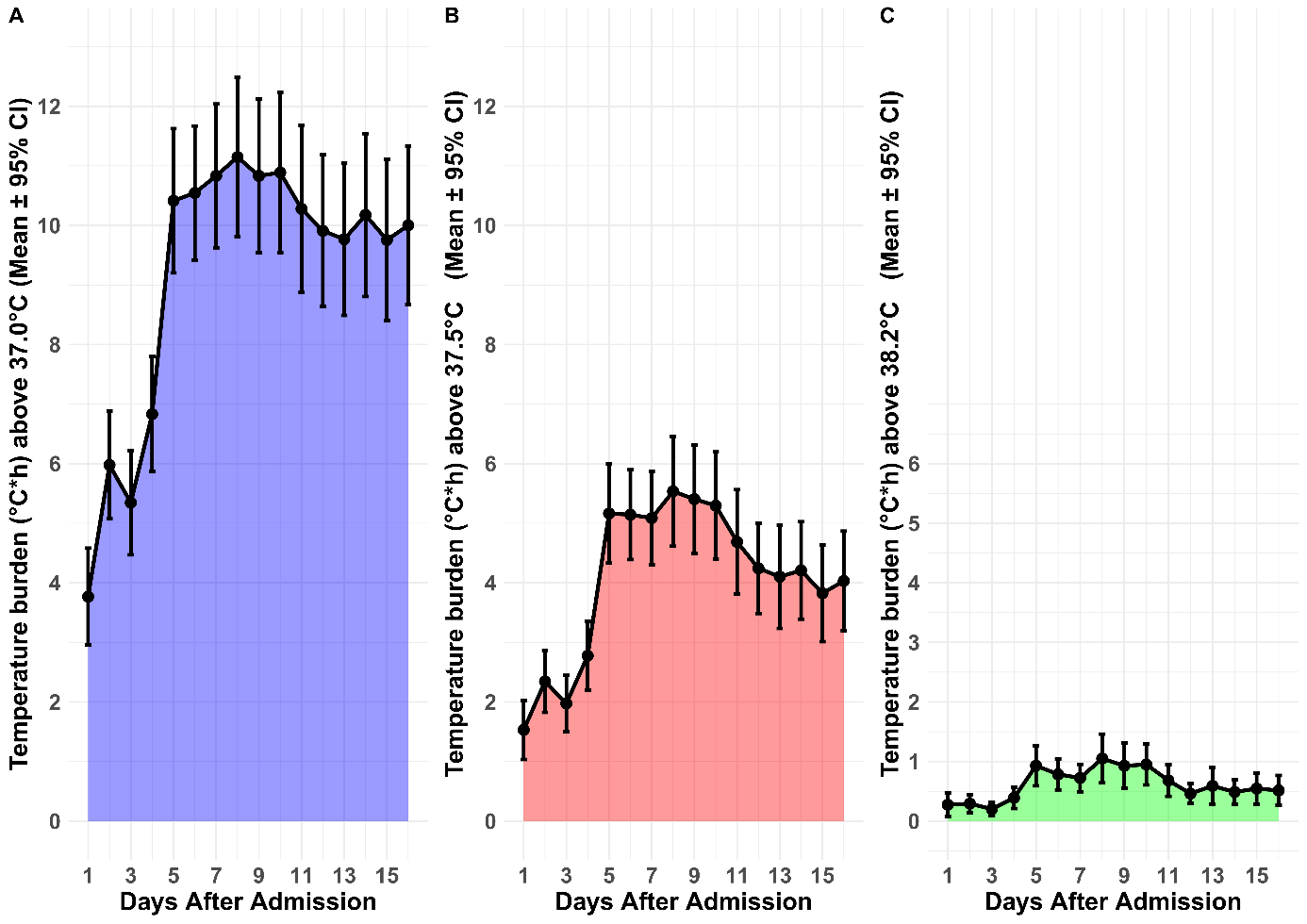


**Supplemental Figure 3:** Average daily temperature burden for (A) >37.0°C, (B) >37.5°C, and (C) >38.2°C. The daily temperature burden, defined as the mean (95%-CI) area over the curve of >37.0°C, >37.5°C, and >38.2°C (= sum of depth of abnormalities multiplied by the hours spent in abnormal temperatures normalized to monitored time) is reported in °C x hours.


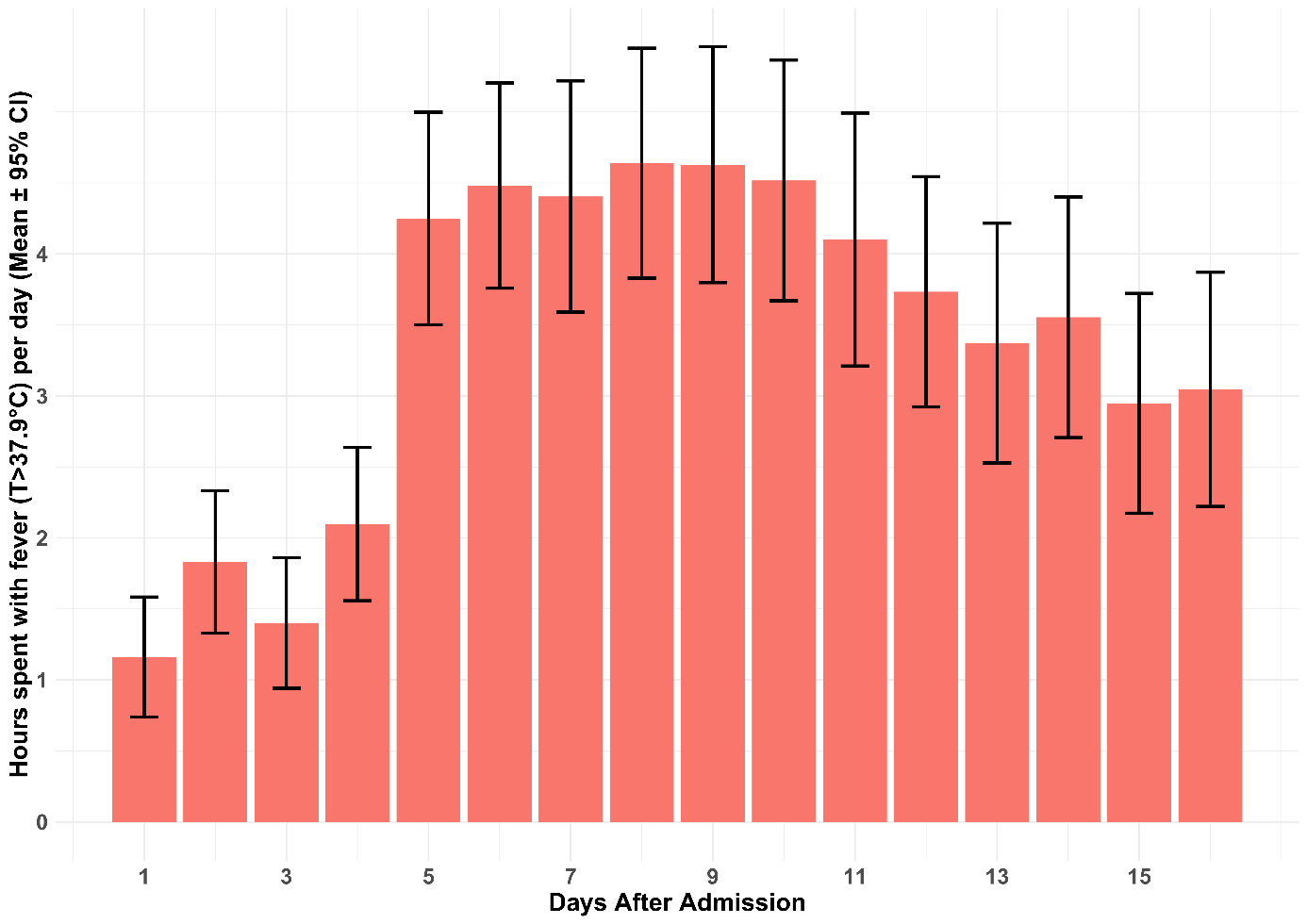


**Supplemental Figure 4:** Time spent in fever (>37.9°C) expressed in daily mean (95%-CI) hours (normalized to monitored time).


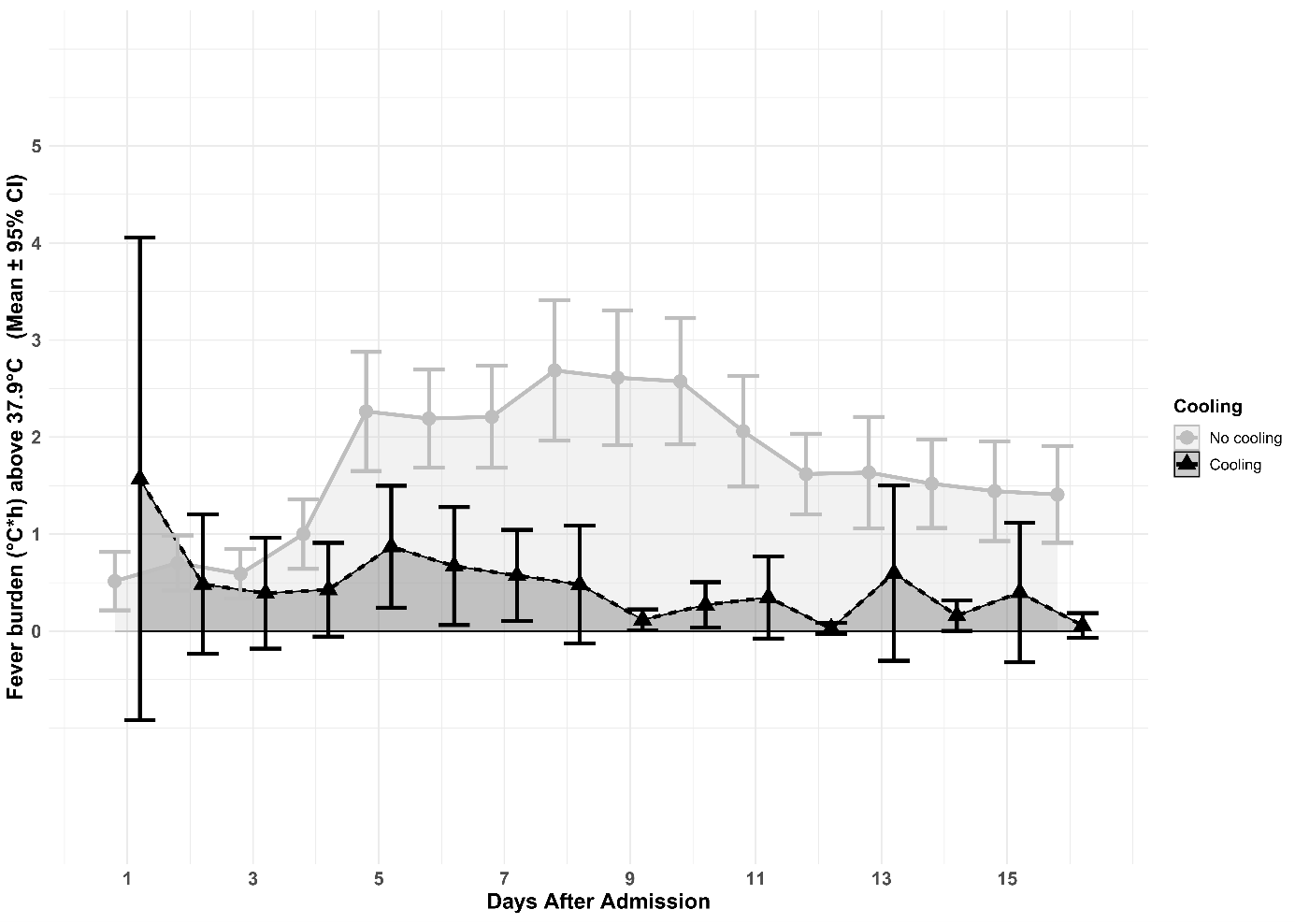


**Supplemental Figure 5:** Average daily fever burden (>37.9°C; in °C x hours) with 95%-CI of the mean for patients with and without a feedback device on respective days.
